# Increasing the nitrate-to-ammonium ratio improved plant growth and nitrogen uptake in pineapple seedlings

**DOI:** 10.3389/fpls.2025.1754688

**Published:** 2026-01-20

**Authors:** Haiyang Ma, Siru Liu, Qiufang Zhao, Yanan Liu, Shuhui Song, Lei Shi, Huayong Li, Weiqi Shi

**Affiliations:** 1 Key Laboratory of Tropical Crop Nutrition of Hainan Province, South Subtropical Crop Research Institute, Chinese Academy of Tropical Agricultural Sciences, Zhanjiang, Guangdong, China; 2 Zhanjiang Comprehensive Scientific Observation and Research Station, Ministry of Agriculture and Rural Affairs, Zhanjiang, Guangdong, China; 3 Guangdong Engineering Technology Research Center of Dryland and Water-Saving Agriculture, South Subtropical Crop Research Institute, Chinese Academy of Tropical Agricultural Sciences, Zhanjiang, Guangdong, China

**Keywords:** growth, nitrogen absorption, NO_3_^-^: NH_4_^+^ ratio, pineapple, root morphology

## Abstract

**Introduction:**

Previous studies indicate that the combined supply of both nitrate nitrogen (NO₃⁻) and ammonium nitrogen (NH₄⁺) is more beneficial for pineapple growth than the exclusive supply of either NO₃⁻ or NH₄⁺ alone. However, the optimal ratio of NO₃⁻:NH₄⁺ that maximizes pineapple growth remains to be fully elucidated, which limits the optimization of nitrogen fertilizer management in pineapple cultivation.

**Methods:**

Uniform plantlets of *Ananas comosus* L. cv. Comte de Paris were subjected to hydroponic experiments with five NO₃⁻:NH₄⁺ ratios (0:100, 30:70, 50:50, 70:30, and 100:0) and a fixed total nitrogen concentration of 4 mM. The shoot and root growth characteristics, as well as carbon (C) and nitrogen (N) accumulation traits of pineapple plants, were systematically investigated. Additionally, the NO₃⁻ and NH₄⁺ uptake rates of roots were determined.

**Results:**

Compared with other treatments, the NO₃⁻:NH₄⁺ ratio of 70:30 significantly increased total root length and surface area, and exhibited the highest NO₃⁻ uptake rate. With increasing nitrate proportions in the nitrogen supply, plant N uptake and the contents of chlorophyll a (Chl a) and chlorophyll b (Chl b) were significantly increased. Specifically, relative to the sole NH₄⁺ treatment (0:100), the 70:30 ratio treatment increased leaf area by 61.10%, leaf dry matter content by 24.40%, plant N accumulation by 19.49%, and plant C accumulation by 21.62%. Enhanced N uptake and photosynthetic pigment synthesis facilitated leaf area expansion, thereby promoting overall plant growth.

**Discussion:**

These results indicate that a NO₃⁻:NH₄⁺ ratio of 70:30 is optimal for pineapple growth, as it effectively enhances both root and shoot development and improves C and N accumulation. This finding provides valuable insights for optimizing nitrogen fertilizer management strategies, which is crucial for promoting pineapple productivity in practical cultivation.

## 1 Introduction

Nitrogen (N) is the nutrient most required by plants and the main component of amino acids, proteins, nucleic acids, chlorophyll, and coenzymes (Marschner, 2012). Ammonium nitrogen (NH_4_^+^) and nitrate nitrogen (NO_3_^-^) are the most available N sources in soil solution to be absorbed by plant roots (Marschner, 2012). Appropriate N form and adequate N supply are the essential basis for optimal plant growth, maximum N use efficiently, and crop yield (Yang et al., 2025). However, the lack of expertise in N management and the unscientific N fertilization practices adopted by farmers resulted in inadequate or excessive amounts of N supply that fail to meet the needs of plant growth. This problem is widespread in agricultural production of crops such as grains, vegetables, and fruits, causing a series of environmental problems (Li et al., 2025). The N absorbed by plants is supplied in organic and inorganic forms, of which ammonium and nitrate are the two major inorganic N forms that can be absorbed and utilized directly from the rhizosphere (Xu et al., 2012; Pereira and Cushman, 2019).

In higher plants, nitrate is delivered to the shoot via xylem after being absorbed into the root and is then converted to ammonium by nitrate reductase (NR) in the cytoplasm and nitrate reductase (NiR) in plastids before being assimilated to synthesize amino acids (Xu et al., 2012). Nitrate reduction is a highly energy-consuming physiological activity, relying on photosynthesis (Pereira and Cushman, 2019; Ortigosa et al., 2020). Direct ammonium absorption would save photosynthetic products and energy, thereby facilitating plant growth (Hachiya and Sakakibara, 2017). Crassulacean acid metabolism (CAM) is characterized by nocturnal CO_2_ uptake and concentration, reduced photorespiration, and increased water-use efficiency when compared to C_3_ and C_4_ plants (Borland and Griffiths, 1989; Yamori et al., 2014; Pereira et al., 2017). Despite the toxicity effects that ammonium showed for most CAM plants, NH_4_^+^ can be advantageous for plant growth due to the low energy requirement needed for its assimilation, when compared to NO_3_^-^ and urea (Pereira et al., 2018; De Andrade-Santos et al., 2020). In contrast, nitrate nitrogen can exist in cells at a high concentration and is quickly transported to other tissues for protein synthesis without damaging plant metabolism; however, the absorption of nitrate nitrogen by roots requires more photosynthetic energy than that of ammonium nitrogen (Coskun et al., 2013; Bittsánszky et al., 2015). The preference and availability for a particular form may vary depending on the form and plant’s ability to assimilate the element into organic compounds (Da Cunha et al., 2024). Previous studies have demonstrated that plant species exhibit distinct preferences for inorganic nitrogen (N) forms. When both ammonium (NH_4_^+^) and nitrate (NO_3_⁻) coexist in the growth environment, most plants display a clear preference for one form over the other. For instance, blueberry and pecan show a tendency to favor NH_4_^+^ (Zhang et al., 2021; Chen et al., 2023), whereas strawberry, Labiatae species, and maize are inclined toward NO_3_⁻ (Tabatabaei et al., 2006; Zhu et al., 2014; Wang et al., 2019). Beyond species-specific preferences, the combined application of NH_4_^+^ and NO_3_⁻ has also been reported to facilitate N uptake and enhance N use efficiency in cherry rootstocks (Xu et al., 2025).

Pineapple (*Ananas comosus* [L.] Merr. var. comosus) is among the five most important tropical fruits in the world. About 90 countries have areas of pineapple cultivation; however, the 10 largest producers account for 70% of total world production (Food and Agriculture Organization of the United Nations, 2023). As a key CAM plant, pineapple growth and productivity heavily depend on nitrogen as an essential macronutrient that directly influences fruit development and yield (Rao et al., 1977; Rodrigues et al., 2014; Darnaudery et al., 2018; Maia et al., 2020; Ma et al., 2022). Previous studies have shown that pineapple probably uses nitrogen in the NH_4_^+^, NO_3_^-^, urea, and glycine forms (Stewart et al., 1925; Sideris et al., 1938; Chen et al., 2018, Chen et al., 2023). Depending on the N form and concentration, the use of sole NH_4_^+^ or NO_3_^-^ supply with NH_4_^+^ solution in 2.86 mmol L^-1^ or NO_3_^-^ solution in 11.43 mmol L^-1^ enhanced the pineapple plant growth; however, other concentrations inhibited the growth of pineapples (Stewart et al., 1925). The growth promotion of pineapple seedlings was also found by foliar application of NH_4_^+^ or amide nitrogen, relative to the foliar application of NO_3_^-^ (Chen et al., 2018). Studies have shown that pineapple (cultivar Perola) is better adapted to NH_4_^+^ than organic nitrogen (Endres and Mercier, 2001). In addition, pineapple absorbs ammonium nitrogen at a significantly higher rate than nitrate with nutrient solutions containing either NH_4_^+^ or NO_3_^-^ as the sole nitrogen source (Sideris et al., 1938). In field-grown pineapple plants, pineapple cultivars preferred to acquire NH_4_^+^, followed by glycine, and the uptake rate of NO_3_^-^ was the lowest, using the stable isotope ^15^N tracer technique (Chen et al., 2023). However, Ravoof (1973) reported that the absorption of NO_3_^-^ from ammonium nitrate culture was higher (60%) than that of NH_4_^+^ (40%), possibly indicating that pineapple plants prefer NO_3_^-^ to NH_4_^+^. Nevertheless, other studies found that mixtures of NH_4_^+^ and NO_3_^-^ were beneficial for plant growth compared to NH_4_^+^ or NO_3_^-^ alone (Roosta and Schjoerring, 2008; Hachiya and Sakakibara, 2017; Wang et al., 2019). Therefore, the specific application ratio of NH_4_^+^ to NO_3_^-^ for pineapple plant growth remains elusive. The present study aimed to investigate the growth and nitrogen assimilation in the pineapple with different NH_4_^+^ and NO_3_^-^ ratios to identify the nitrogen management capable of increasing the growth response. We hypothesize that low ammonium with high nitrate concentration supply promotes nitrogen absorption and stimulates pineapple growth. Specifically, we investigated the effects of different NH_4_^+^ and NO_3_^-^ ratios in constant levels of N on plant growth, photosynthetic pigments, root morphology, and carbon and nitrogen accumulation. The findings will provide valuable insights into potential strategies of N management for sustainable pineapple production.

## 2 Materials and methods

### 2.1 Plant growth conditions

Plantlets of *Ananas comosus* L. cv. Comte de Paris with uniform weight and appearance (approximately 100 g in weight and 15 cm in height) were selected. Subsequently, the selected plantlets were transferred to 1/2-strength modified Hoagland solution (pH 5.5) for a 45-day hydroponic pre-culture, with the solution renewed weekly. After pre-culture, plantlets with uniform growth were selected for the nutrient uptake assay. The plantlets were subjected to different NO_3_^-^: NH_4_^+^ ratio treatments under hydroponic conditions. The nutrient solutions were composed of 4 mM nitrogen (with different NH_4_^+^:NO_3_^-^ ratio), 1.0 mM KH_2_PO_4_, 2.0 mM K_2_SO_4_, 2.0 mM MgSO_4_·7H_2_O, 0.1 mM CaCl_2_·2H_2_O for macro-elements, and 10 µM MnSO_4_·4H_2_O, 10 µM ZnSO_4_·7H_2_O, 1.0 µM CuSO_4_·5H_2_O, 50µM H_3_BO_4_, 0.5 µM Na_2_MoO_4_·2H_2_O, 0.2 µM CoSO_4_·7H_2_O, 0.1 mM NaCl and 50 µM Fe-Na-EDTA·2H_2_O for micro-elements and Ca(NO_3_)_2_ for the nitrate source or NH_4_Cl for the ammonium source (Sideris et al., 1938). The NO_3_^-^: NH_4_^+^ ratio treatments were 0:100, 30:70, 50:50, 70:30, and 100:0. Each treatment contained 4 replicates. Each replicate included one plant grown in 700 mL of nutrient solution. The pH of the nutrient solution was maintained at pH 5.5 ± 0.1 with 1 mmol·L^-1^ HCl or NaOH. The nutrient solution was renewed every three days. The room temperature was 28°C, the relative humidity was 80%, the photosynthetic photon flux density was 500 µmol·m^-2^·s^-1^, and the photoperiod was 8h/16h (day/night). The plants were harvested after three months of treatment.

### 2.2 Ammonium and nitrate uptake rates

The pre-cultured pineapple plantlets as detailed in Section 2.1 were subjected to a 3-day nitrogen starvation treatment, with the culture medium substituted with pure water. Following nitrogen starvation, the roots were soaked in 0.1 mM CaSO_4_ for 5 minutes to exchange the ions adhering to them. Following rinsing three times with reverse osmosis water (NO_3_⁻ ≤ 0.1 mg·L-1), the roots were immersed in the nutrient solutions corresponding to the aforementioned hydroponic treatments, which contained a fixed total nitrogen concentration of 4 mM with varying NO_3_^-^:NH_4_^+^ ratios (0:100, 30:70, 50:50, 70:30, 100:0) and were supplemented with nitrate as Ca(NO_3_)_2_ and ammonium as NH_4_Cl. Each pineapple seedling was carefully placed in a 600 mL absorption bottle for an hour from 9:00 to 10:00, with its roots submerged in either 500 mL of nutrient solution. The exterior of the bottle was covered with tin foil to prevent light from affecting the roots. After one hour of reaction, the residual fluid was collected, and the fresh weight of the corresponding roots was measured. The concentrations of ammonium and nitrate retained in the uptake solution were determined using Nessler’s reagent spectrophotometry and the sulfonamide colorimetric method, according to the manufacturer’s instructions, respectively. The ammonium absorption rate was calculated as,

Nitrogen absorption rate = (initial concentration - sample concentration) × volume/(absorption time × root weight).

### 2.3 Leaf growth, photosynthetic pigment, root morphology, and activity

The D leaf is defined as the youngest physiologically mature leaf on the plant and is also the tallest leaf on the plant. The D leaf width was measured in the median region of the leaf. The leaf area was measured with a laser area meter (model CI-203, CID Bio-Science, WA, USA). Determination of the photosynthetic pigments content followed the method described by Li et al. (2000).

The root morphology was determined by WinRHIZO Pro (Regent Instruments, QC, Canada) after the root images were scanned with a scanner (Epson Expression 12000XL, Seiko Epson Corporation, Japan). The total root length, root surface area, root volume, and average root diameter were obtained. Fresh and white root samples of each plantlet were collected at the end of the treatment. The root activity was determined through the 2, 3, 5-triphenyltetrazolium chloride method (TTC) using detection kits (Suzhou Comin Biotechnology Co. Ltd., Suzhou, China) following the manufacturer’s protocols.

### 2.4 Carbon and nitrogen concentration

At the end of the treatment, the root was soaked in a 0.1 mM CaSO_4_ solution for 5 minutes to exchange the ions adsorbed on the roots. Then, the roots and shoots were harvested. After drying at 105°C for 30 minutes, the shoot and root were dried at 80°C to constant weight. The dry biomass was ground using a ball mill and then passed through a 0.425 mm sieve. The carbon and nitrogen concentrations were determined by an element analyzer (vario PYRO cube, Elementar Analysensysteme GmbH, Germany).

### 2.5 Statistical analysis

Statistical analyses were performed using SPSS 25.0, and the differences among different treatments were analyzed using one-way analysis of variance (ANOVA) followed by Duncan’s *post hoc* multiple comparisons tests (P<0.05). Figures were drawn by Origin 2021.

## 3 Results

### 3.1 Leaf growth

**Figure 1:**
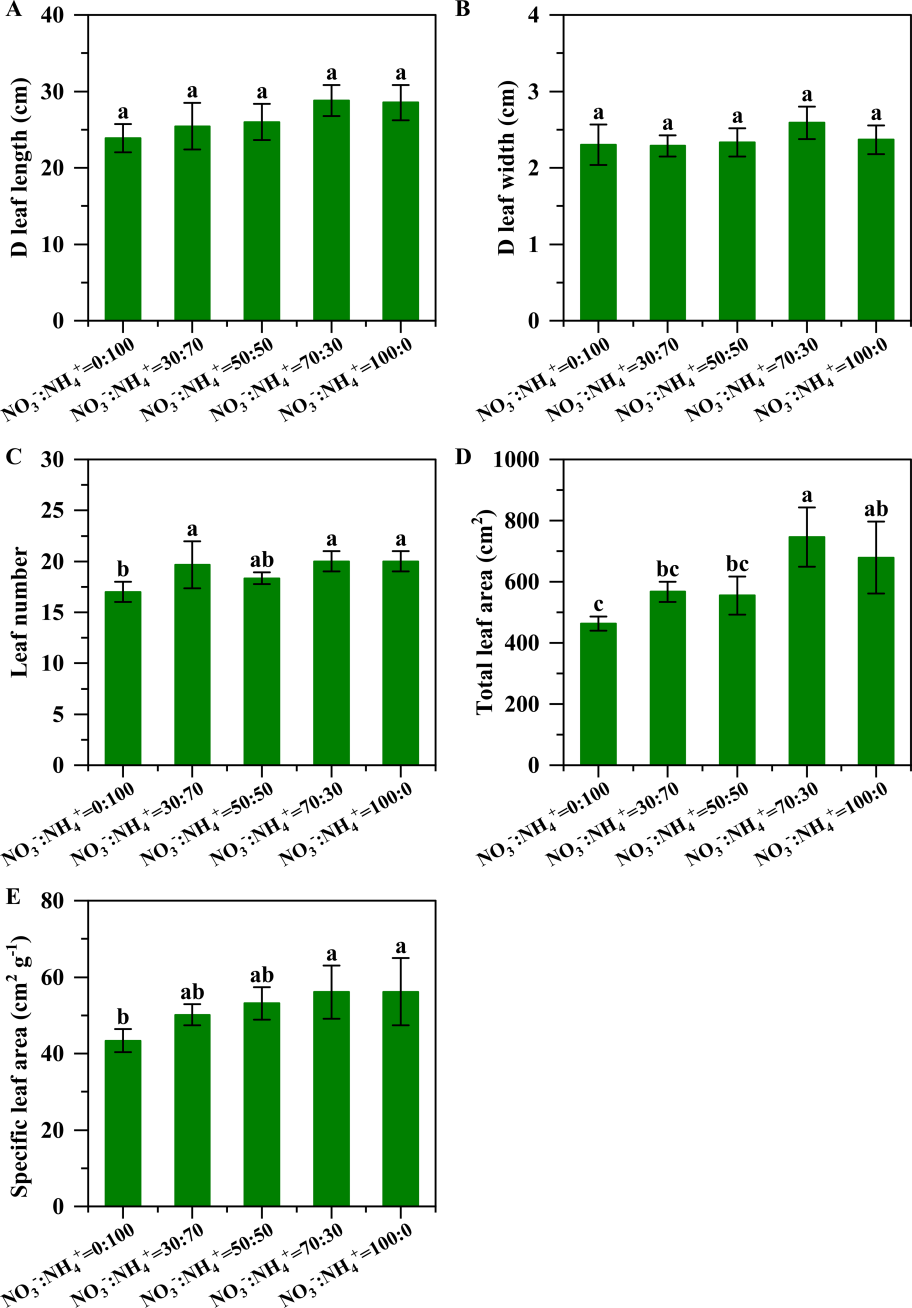
Leaf growth of pineapple under different NO_3_^-^: NH_4_^+^ ratio treatments. The D leaf length **(A)**, D leaf width **(B)**, leaf number **(C)**, total leaf area **(D)**, and specific leaf area **(E)**. The different letters above the columns represent significant differences between treatments (P <0.05).

The NO_3_^-^: NH_4_^+^ ratios significantly affected the total leaf area of pineapple (**Figure 1D**). With the increase of nitrate ratio in the nutrition solution culture, the leaf area increased initially and then decreased. The maximum leaf area was found in the treatment with NO_3_^-^: NH_4_^+^ ratio of 70:30 (**Figure 1D**). Compared with the supply of ammonium nitrogen alone, the leaf area increased by 22.36%, 19.81%, 61.10% and 46.52% (**Figure 1D**) in the 30:70, 50:50, 70:30, and 100:0, respectively. In addition, the NO_3_^-^: NH_4_^+^ ratio had a significant influence on the leaf number (**Figure 1C**) and the specific leaf area (**Figure 1E**), but it showed no significant effect on the D leaf length and width of pineapple (**Figures 1A, B**). Compared with the supply of sole ammonium nitrogen, the leaf number increased by 15.69%, 7.84%, 17.65% and 17.65% (**Figure 1C**), and the specific leaf area increased by 15.63%, 22.49%, 29.29% and 29.53% (**Figure 1E**) in the 30:70, 50:50, 70:30, and 100:0, respectively.

### 3.2 Leaf photosynthetic pigments

**Figure 2:**
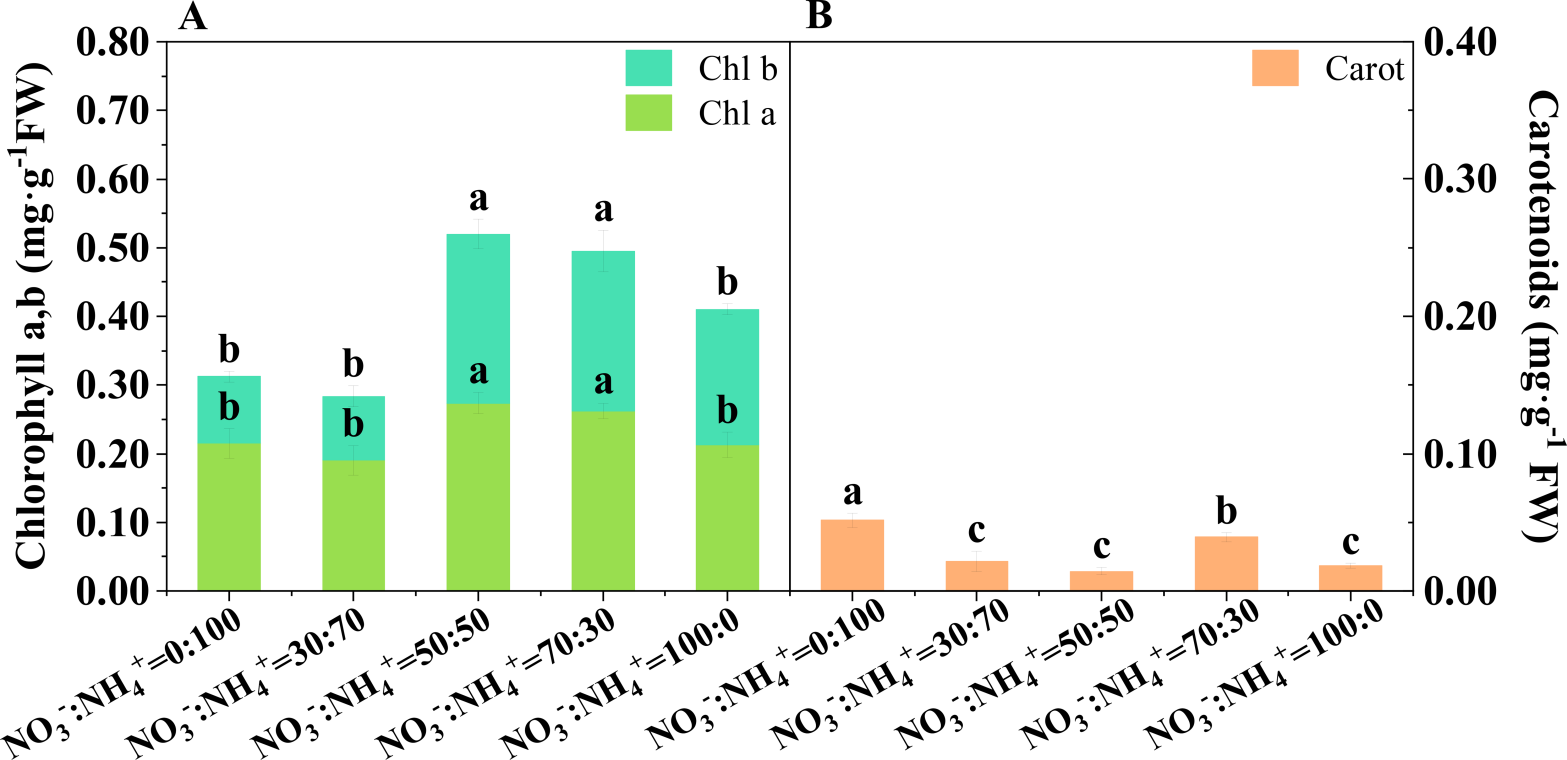
Photosynthetic pigments concentration in the D leaves of *Ananas comosus* var. comte de Paris for three months with different NO_3_^-^: NH_4_^+^ ratio treatments. The chlorophyll a and b **(A)** and carotenoids **(B)**. Different letters indicate significant statistical differences (P<0.05).

The contents of chlorophylls a and b showed an upward trend with increasing nitrate proportions (**Figure 2A**). The NO_3_^-^: NH_4_^+^ ratios of 50:50 and 70:30 significantly increased the contents of chlorophyll a and b. The content of carotenoids was the highest under the sole ammonium treatment, followed by the treatment with NO_3_^-^: NH_4_^+^ ratio of 70:30, compared with other treatments (**Figure 2B**).

### 3.3 Root morphology and activity

**Figure 3:**
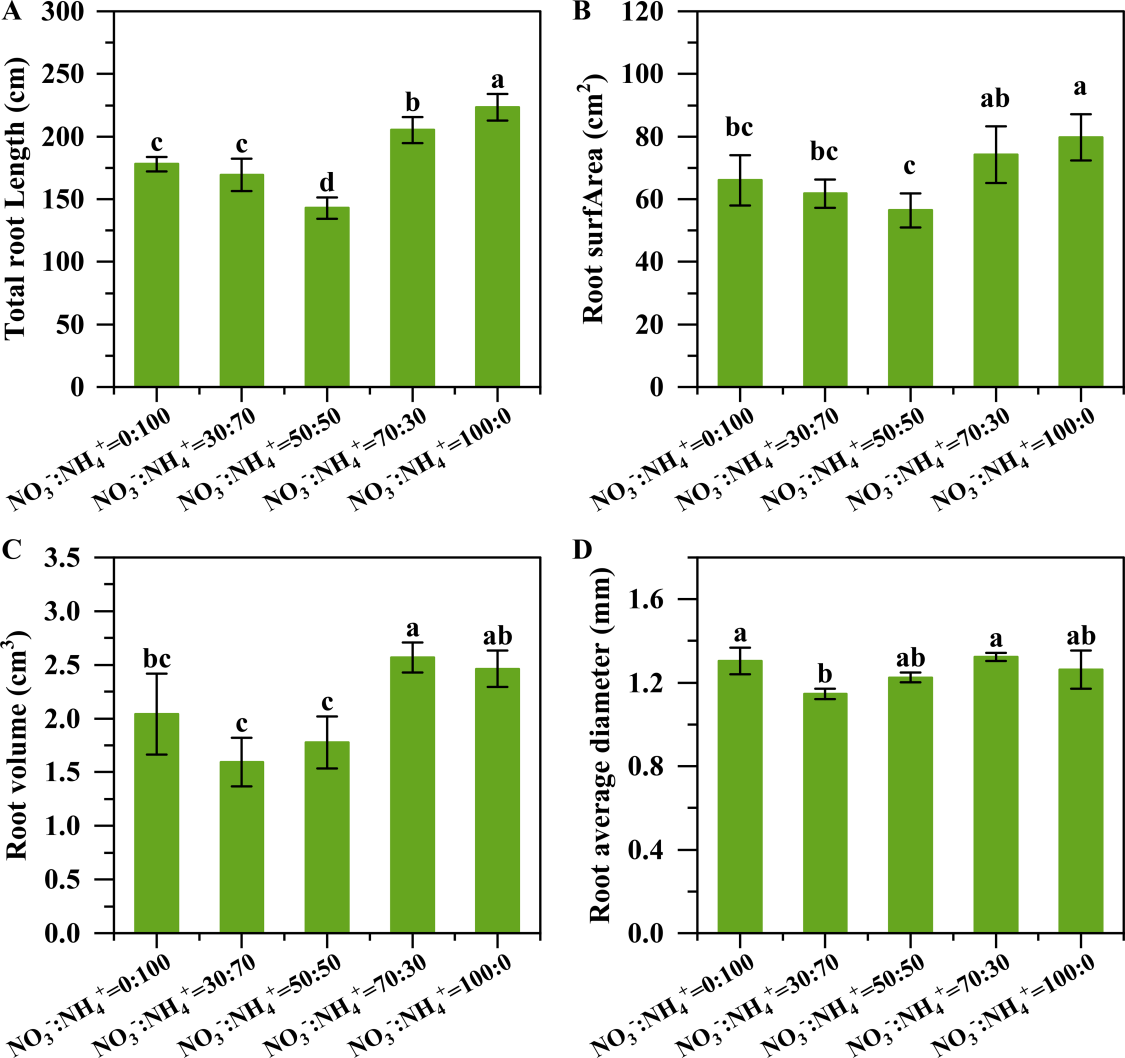
Root morphology of pineapple under different NO_3_^-^: NH_4_^+^ ratio treatments. The root total length **(A)**, root total surface area **(B)**, root volume **(C)**, and root average diameter **(D)**. Different lowercase letters above the columns represent significant differences between treatments (P<0.05).

**Figure 4:**
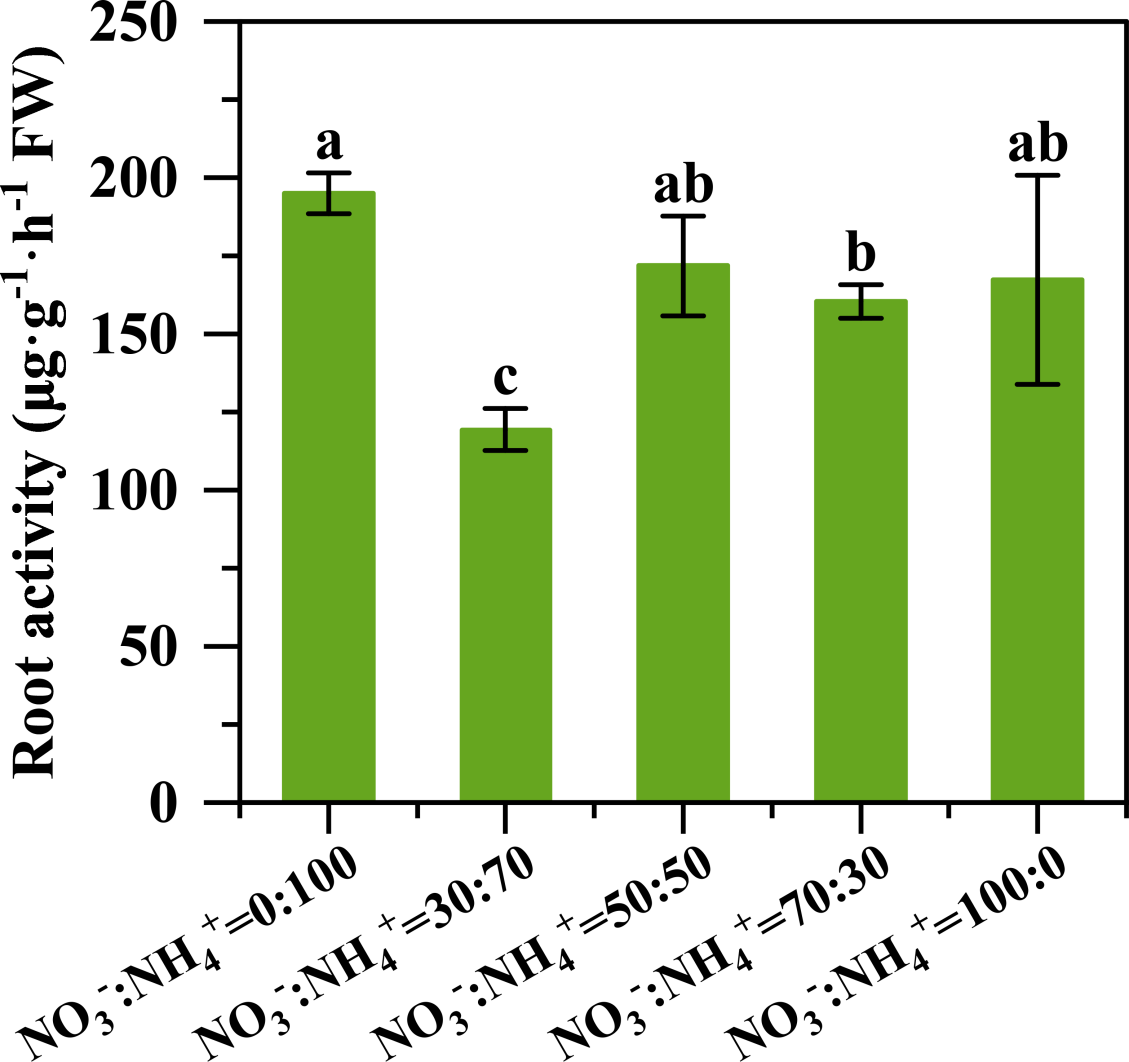
Root activity of the pineapple plant three months after different NO_3_^-^: NH_4_^+^ ratio treatments. Columns marked with different lowercase letters indicate significant differences (P < 0.05) between treatments based on the Duncan test. Dates are shown as the mean values ± standard deviation (SD) (N = 4).

With the increase of nitrate proportions, the length, surface area, volume, and average diameter of the root firstly decreased and then increased (**Figure 3**). The maximum root volume and average diameter were observed under the NO_3_^-^: NH_4_^+^ ratio of 70:30. Compared to the treatment with the supply of only ammonium nitrogen, the root length changed by -4.85%, -19.68%, 15.32% and 25.58% (**Figure 3A**), the total root surface area varied by -6.47%, -14.56%, 12.35% and 20.73% (**Figure 3B**), the total root volume altered by -21.80%, -12.81%, 25.83% and 20.76% (**Figure 3C**), and the total root average diameter modified by-12.06%, -6.05%, 1.59% and -3.14% (**Figure 3D**) with the increase of nitrate proportion to 30%, 50%, 70%, and 100%, respectively. With the decrease of the ammonium ratio, the root activity of pineapple was obviously decreased and then increased, and the root activity was highest when the ammonium was supplied alone (**Figure 4**).

### 3.4 Ammonium and nitrate uptake rates

**Figure 5:**
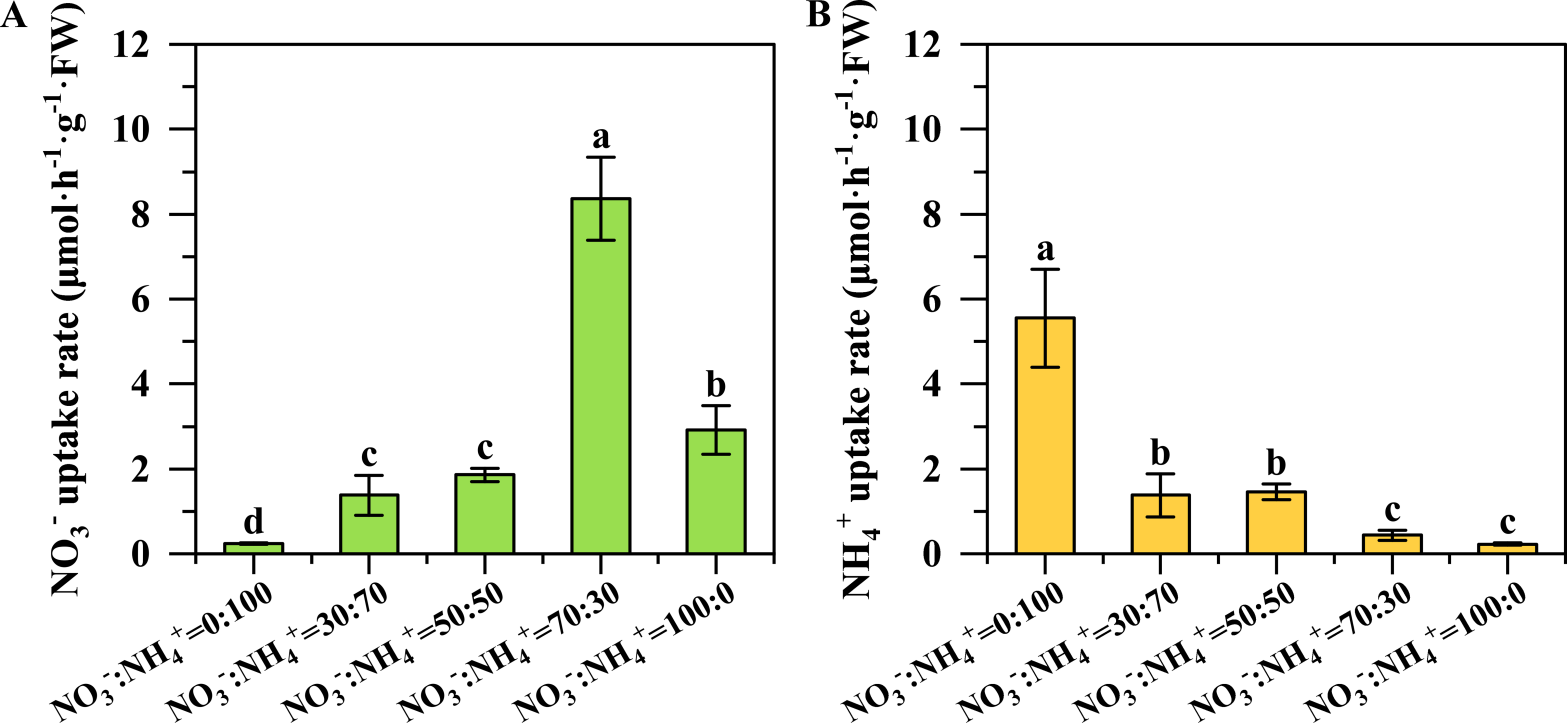
Ammonium and nitrate uptake rates in pineapple under different NO_3_^-^: NH_4_^+^ ratio treatments. Nitrate uptake rate **(A)**, and ammonium uptake rate **(B)**. N=4. The different letters above the columns represent significant differences between treatments (P < 0.05).

With the decreasing proportion of ammonium, the absorption rates of ammonium decreased. However, the absorption rates of nitrate first increased and then decreased. The maximum absorption rate of nitrate was observed under the NO_3_^-^: NH_4_^+^ ratio of 70:30 (**Figure 5A**), while the highest absorption rate of ammonium was found in the only ammonium supplied treatment (**Figure 5B**).

### 3.5 Biomass accumulation

**Figure 6:**
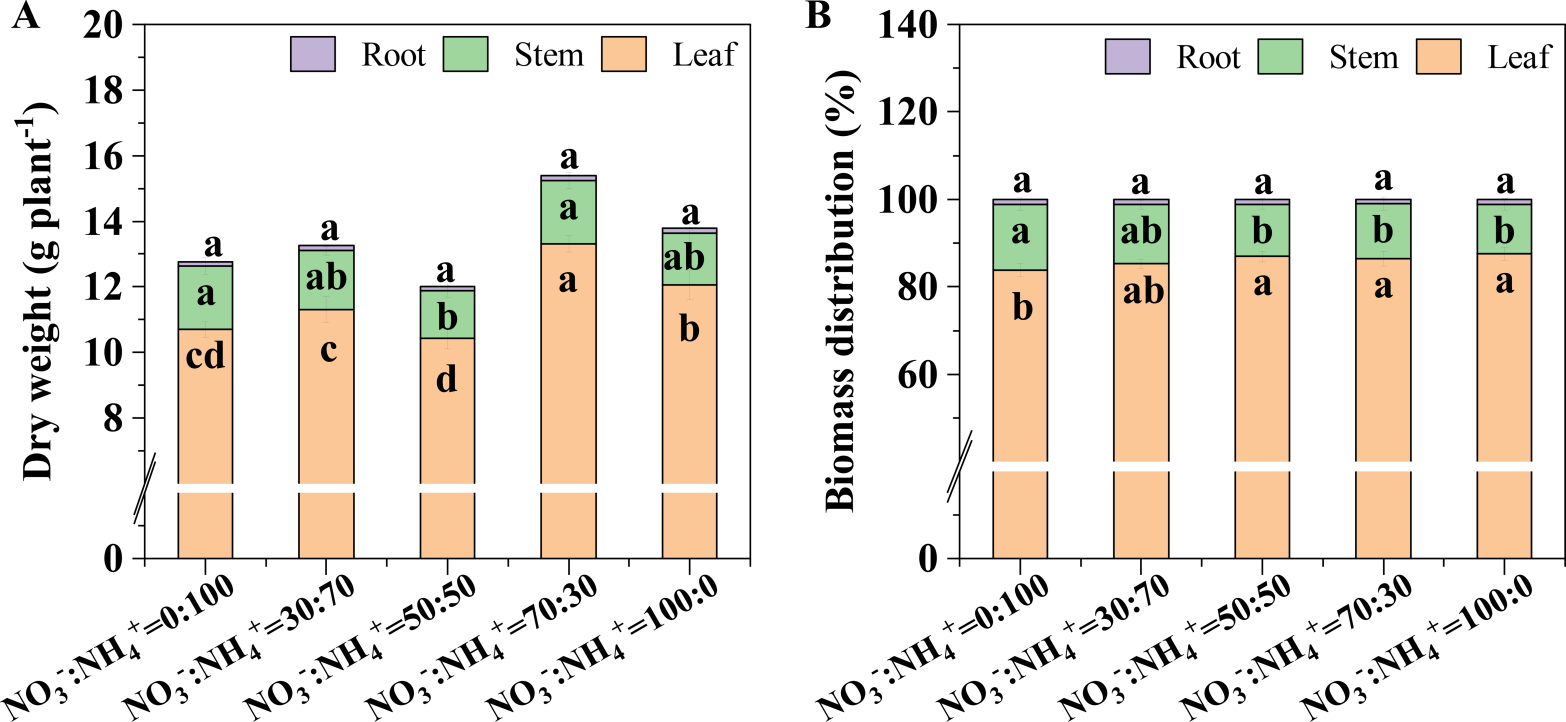
The biomass accumulation and distribution in pineapple that received three months of different NO_3_^-^: NH_4_^+^ ratio treatments. The dry weight **(A)** and biomass distribution **(B)**. N = 4. The different letters above the columns represent significant differences between treatments (P<0.05).

With the increase of nitrate proportion, the biomass firstly decreased and then increased. The highest dry weights of pineapple leaf and stem were noted in the NO_3_^-^: NH_4_^+^ ratio of 70:30 treatment (**Figure 6A**). Pineapple in the treatment with the NO_3_^-^: NH_4_^+^ ratio of 50:50 showed the lowest dry weight of leaf and stem. Significantly more dry matter was partitioned into the shoots with the increase of nitrate proportion (**Figure 6B**).

### 3.6 Plant C and N concentration and accumulation

**Figure 7:**
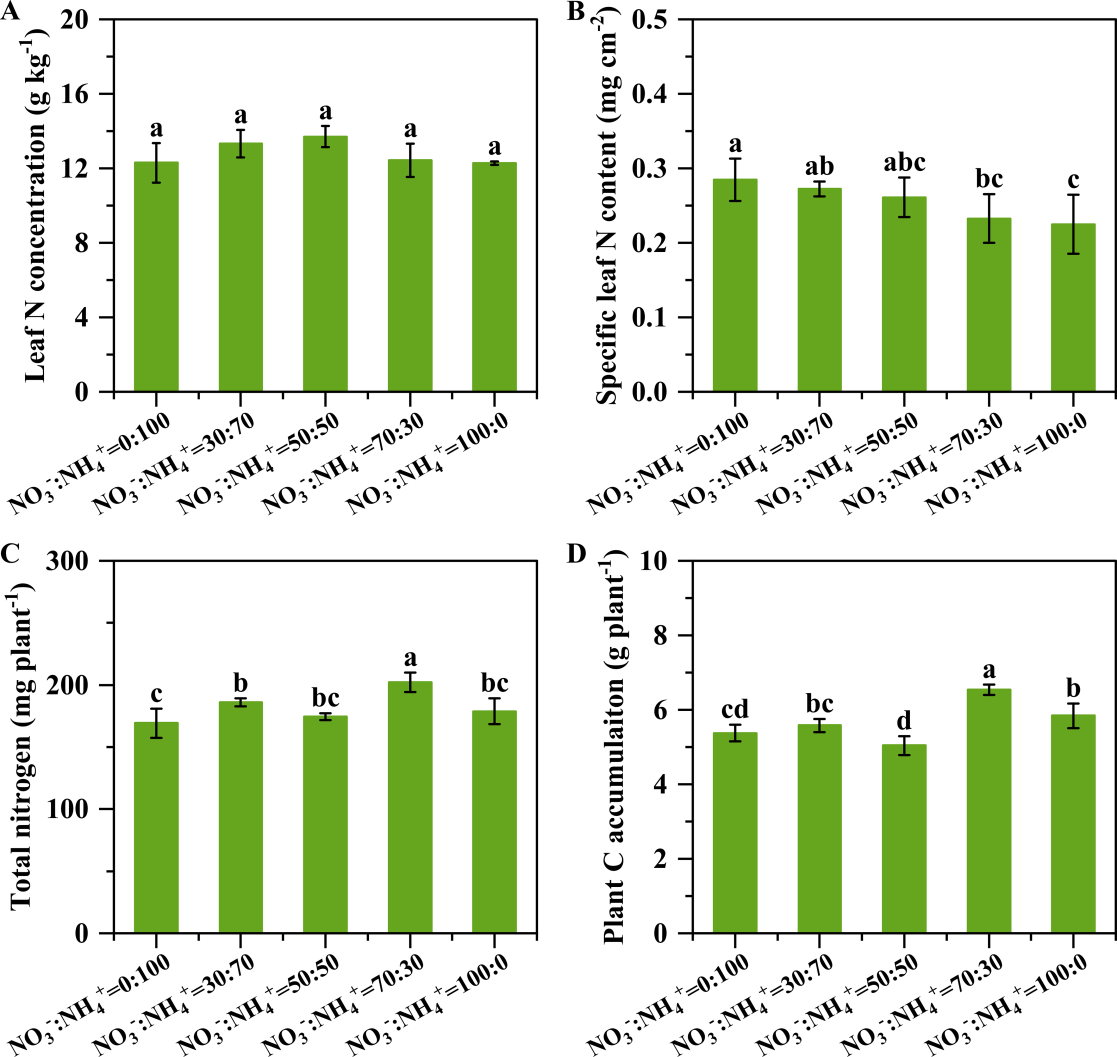
Leaf carbon and nitrogen concentration and accumulation in pineapple that received three months of different NO_3_^-^: NH_4_^+^ ratio treatments. The leaf N concentration **(A)**, specific leaf N content **(B)**, total nitrogen accumulation **(C)** and plant C accumulation **(D)**. N = 4. The different letters above the columns represent significant differences between treatments (P<0.05).

**Figure 8:**
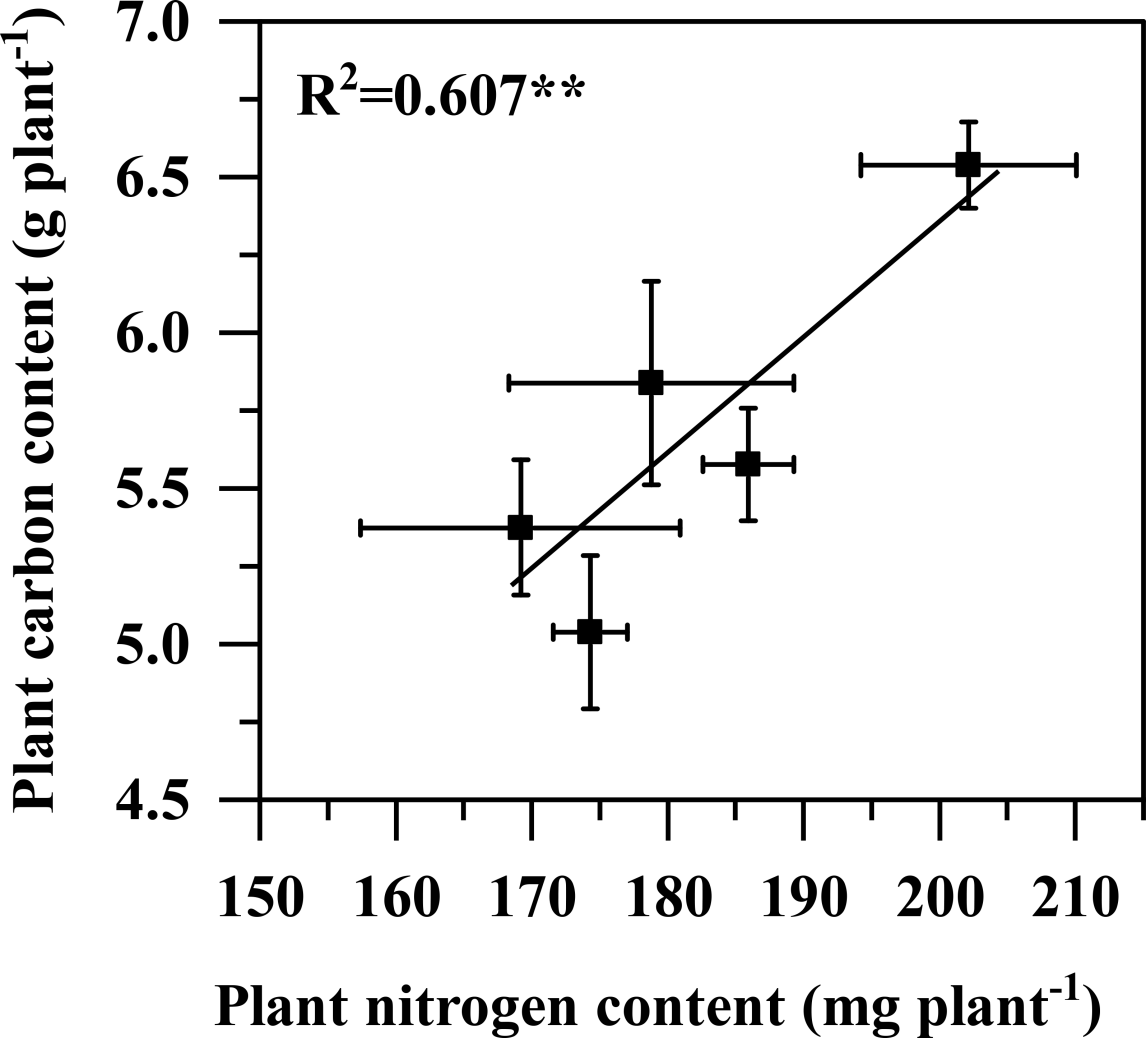
Relationships between plant carbon and plant nitrogen accumulation of pineapple plants that received three months of different NO_3_^-^: NH_4_^+^ ratio treatments. The regression line is for the data in all treatments. ** indicate significance of the regression line at P < 0.01.

The leaf N concentration was similar among all the treatments (**Figure 7A**). However, the specific leaf N content of pineapple plants decreased with the increasing nitrate proportion, with the lowest specific leaf N content found in the only nitrate treatment (**Figure 7B**). The carbon and nitrogen accumulation firstly decreased and then increased with increasing nitrate proportions (**Figures 7C, D**). The treatment with NO_3_^-^: NH_4_^+^ ratio of 70:30 had the maximum carbon and nitrogen accumulation. Compared with only ammonium treatment, increasing the nitrate proportions improved the nitrogen accumulation by 9.93%, 3.06%, 19.49% and 5.68% (**Figure 7C**), and the carbon accumulation by 3.77%, -6.26%, 21.62% and 8.63% (**Figure 7D**), respectively. There was a significant positive linear relationship between the nitrogen and carbon accumulation (**Figure 8**).

## 4 Discussion

Appropriate NO_3_^-^: NH_4_^+^ ratios promoting plant growth has been reported for various plant species such as the optimal NO_3_^-^: NH_4_^+^ ratio of 17: 83 for blueberry (Zhang et al., 2021); 25: 75 for pecan (Chen et al., 2023); 50:50 for coffee (Carr et al., 2020) and yellow passion fruit (Da Cunha et al., 2024) as well as 75:25 for strawberry, Labiatae and maize (Tabatabaei et al., 2006; Zhu et al., 2014; Wang et al., 2019). In this study, the NO_3_^-^: NH_4_^+^ ratio of 70:30 treatment achieved the highest biomass, carbon, and nitrogen accumulation, showing a significant improvement compared with other ratios involving the sole application of either ammonium or nitrate (**Figure 6**).

The NO_3_^-^: NH_4_^+^ ratio of 70:30 treatment improved the root growth and nitrate uptake ability, contributing to enhancing nitrogen assimilation. The root morphology plays an essential role in nitrogen uptake (Meister et al., 2014). Numerous studies have shown that an appropriate NO_3_^-^: NH_4_^+^ ratio can promote the formation of better root morphology (Tabatabaei et al., 2006; Hu et al., 2017; Liu et al., 2017; Wang et al., 2019; Carr et al., 2020; Zhang et al., 2021). Our results revealed that when the proportion of nitrate nitrogen exceeded 50%, the total root length and surface area of pineapple plants increased significantly (**Figure 3**), leading to a larger absorption area, thereby enhancing nitrogen uptake. Such responses were confirmed again by the leaf N concentration that was not reduced by the dilution effect, but significantly higher plant N and C accumulation (**Figures 7C, D**). Nutrient uptake of plants is determined by both the root system that can contact the nutrients and the root assimilation ability. In the current study, we found that pineapple roots had the strongest NO_3_^-^ absorption rate when supplied with the NO_3_^-^: NH_4_^+^ ratio of 70: 30, which consequently promoted nitrogen uptake under this treatment. It was noteworthy that the NH_4_^+^ absorption rate decreased significantly along with the decreasing NH_4_^+^ ratios (**Figure 5**). A previous study with maize noted the maximum nitrate absorption rate under the NO_3_^-^: NH_4_^+^ ratio of 75: 25 (Wang et al., 2019; Yang et al., 2025).

The improved plant nitrogen uptake due to increased root growth and enhanced root NO_3_^-^ absorption rate of pineapple can enhance the synthesis of chlorophylls and carotenoids (Bassi et al., 2018; Wen et al., 2019). In the current study, the content of chlorophyll a and b in the leaves of the NO_3_^-^: NH_4_^+^ ratio of 70:30 supply treatment was significantly higher than that of the ammonium and nitrate supply treatments alone. Thus, the photosynthetic capacity of leaves is expected to be significantly enhanced, enabling the synthesis of more sugars (Bassi et al., 2018).

The increased root growth and N uptake rate can facilitate leaf expansion. This study observed that the leaf area of the pineapple plants in the NO_3_^-^: NH_4_^+^ ratio of 70:30 supply treatment significantly increased by 61.10%, whereas the supply of ammonium alone inhibited the growth of leaves (**Figure 1D**). Plant leaf area is a key factor determining the dry matter accumulation of pineapples, as it is directly related to photosynthesis and photosynthate fixation (An et al., 2022). Corresponding to this leaf area enlargement, the leaf dry matter content was significantly enhanced by 24.40% in this treatment (**Figure 6A**), which aligned with the positive correlation between leaf area and dry matter accumulation.

Different NH_4_^+^ and NO_3_^-^ ratios significantly altered the growth and nitrogen assimilation of pineapple in hydroponic systems, these variations further affected plant’s nutrient uptake efficiency. It is well-documented that soil N transformation processes (e.g., nitrification, denitrification) and associated microbial activities regulate nitrogen availability in field environments, thereby mediating plant nitrogen acquisition (Zhang et al., 2018; Fu et al., 2025). However, our current study did not measure these soil N transformation dynamics or account for the role of microbial activities, as the hydroponic system lacks the soil matrix required for such processes. Therefore, future research should focus on validating our findings through pot experiments using field soils and *in situ* field trials. These studies should explicitly incorporate analyses of nitrogen cycling dynamics to clarify how soil-specific factors modulate the effects of NH_4_^+^/NO_3_⁻ ratios on plant performance, thereby enhancing the practical applicability of our results.

## 5 Conclusions

Supplying a low ammonium with a high nitrate concentration promoted nitrogen absorption and stimulated pineapple growth. Pineapple plants grown with NO_3_^-^: NH_4_^+^ ratio of 70:30 exhibited the highest biomass, as well as carbon and nitrogen accumulation, compared with other treatments (including the sole ammonium, sole nitrate, and other mixed nitrogen ratios). Specifically, the NO_3_^-^: NH_4_^+^ ratio of 70: 30 enhanced pineapple root growth and nitrate uptake ability, thereby promoted nitrogen assimilation. Furthermore, the concentrations of chlorophyll a and b in leaves of the NO_3_^-^: NH_4_^+^ ratio of 70:30 treatment were significantly higher than those in the sole ammonium and sole nitrate treatments, indicating enhanced photosynthetic capacity of pineapple leaves and thereby facilitating the synthesis of more sugars. Consequently, leaf area expansion in pineapple plants under the NO_3_^-^: NH_4_^+^ ratio of 70:30 treatment was promoted. Corresponding to this leaf area enlargement, the leaf dry matter content in this treatment was significantly increased by 24.40%. The results suggest 70:30 as the optimum NO_3_^-^: NH_4_^+^ ratio, which can enhance the root and shoot growth, and C and N accumulation of pineapple.

## Data Availability

The original contributions presented in the study are included in the article/supplementary material. Further inquiries can be directed to the corresponding authors.
